# Supplementary material for: How face perception unfolds over time
Source: Nat Commun. 2019 Mar 19;10:1258. doi: 10.1038/s41467-019-09239-1 (PMC6425020; doi:10.1038/s41467-019-09239-1)
Supplement: Supplementary file 1 — Supplementary Information [file 41467_2019_9239_MOESM1_ESM.pdf]

# How face perception unfolds over time

Supplementary Information

Dobs et al.

**Supplementary Note 1: Stimulus-driven familiarity effects in a deep neural network**

**Methods.** Our MEG analysis of familiarity effects is based on different sets of face stimuli consisting of American and German celebrities, respectively. To control for low-level differences in the different sets of stimuli, we partialled out low-level features based on the second convolutional layer of CNN trained on faces, namely VGG-Face, in our main analysis. However, despite controlling for low-level features, it is still possible that some face dimensions, such as gender, are more discriminable in one image set compared to the other. To test this, we used VGG-Face as a generalized face recognition model [1] and investigated whether an early or late layer in this model would show stimulus-driven differences between the American (familiar to our subjects) and German (unfamiliar to our subjects) celebrity stimuli for any of the face dimensions. Specifically, in addition to the second convolutional layer, we extracted features for all our face stimuli from the last fully connected layer of VGG-Face. We used 1 – Pearson correlation as a measure of dissimilarity between the units of each pair of stimuli, resulting in a 80 x 80 RDM based on low- and high-level face representations in this CNN. We tested for any differences between the American versus German celebrity stimulus sets by dividing the CNN and model RDMs (i.e., gender, age and identity) into within American and within German celebrity RDMs, respectively. Each of these RDMs consisted of a 40 x 40 RDM containing pairwise differences of familiar or unfamiliar face images, respectively. As for the MEG analyses, for each model (e.g., gender), we partialled out all the other models (e.g., age and identity) and, in case of the late layer, additionally the low-level feature model. Note that while partialling out the low-level feature did not substantially affect the results, our motivation was to keep the analysis as similar as possible to the MEG analysis. We then computed confidence intervals by bootstrapping the images of the RDMs 1000 times. To test for differences between the American versus German celebrity stimulus sets, we used two-sided bootstrap tests ($p < 0.05$) and FDR correction. Because VGG-Face has not been trained on any of our stimuli, and thus is unfamiliar with all images, any difference in the representations between American and German celebrity stimuli will be due to low-level (in case of the early layer) or high-level (in case of the late layer) stimulus differences in the selected image sets.

**Results.** In the early layer, we found that none of the models significantly correlated with the feature representations in this layer, and we did not find any differences between the face dimensions for American versus German celebrity images (Supplementary Fig. 1a; all $p > 0.05$; two-sided bootstrap test, FDR-corrected). Interestingly, for the late layer the model correlations

increased dramatically (Supplementary Fig. 1b; all correlations above chance), even for gender and age which VGG-Face has not been trained on, again supporting the use of CNNs trained of face identity as generic models for face processing. We did find some differences between our American and German celebrity faces in the late layer. First, we find that age can be discriminated significantly better for American than German face images ($p < 0.01$). While this finding suggests that our American face stimuli (familiar to our subjects) contain more age information than our German face stimuli (unfamiliar to our subjects), this effect is not reflected in our MEG data, in which we did not find a significant difference in the ability to discriminate age between familiar and unfamiliar faces. Another small but significant difference was found between the VGG-Face identity representations of German and American faces ($p < 0.05$). However, this effect showed the opposite trend of what we found in the MEG data: higher discriminability of identity for German than for American celebrity faces. Overall, while the results of this analysis shows that some differences in the discriminability of high-level face dimensions are present between the American and German stimulus sets, these differences cannot straightforwardly explain the strong familiarity enhancement effect for gender and identity for the MEG data.

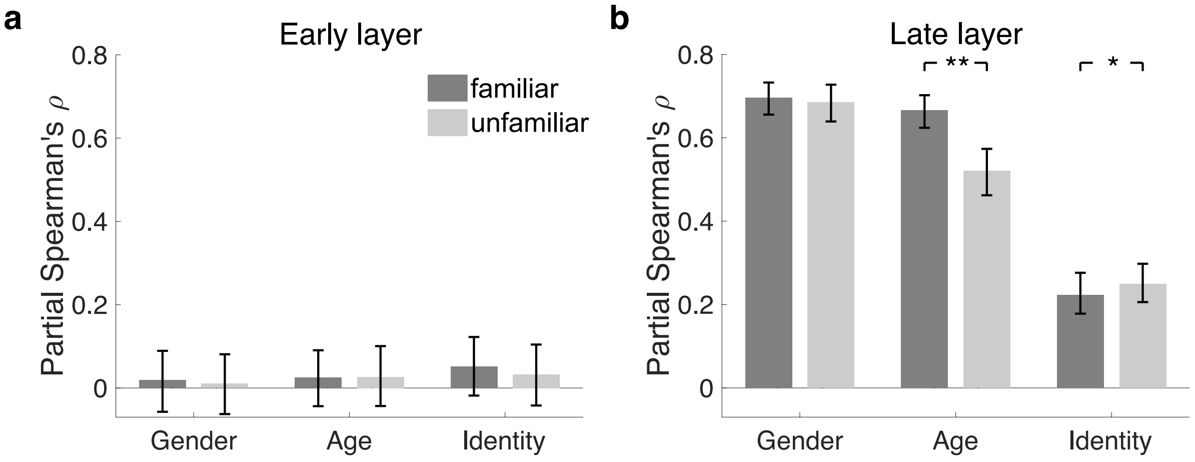


**Supplementary Figure 1 | Analysis of familiarity effects in VGG-Face.** (**a**) Partial Spearman correlations between RDMs of an early (second convolutional) layer of VGG-Face and gender, age or identity model RDMs separated for familiar (dark gray) and unfamiliar (light gray) faces, while partialling out all other models. (**b**) Partial Spearman correlations between RDMs of a late (last fully connected) layer of VGG-Face and gender, age and identity model RDMs separated for familiar and unfamiliar faces, while partialling out other models and an early layer of VGG-Face. Error bars indicate bootstrapped 95% confidence intervals across images (n = 80). Stars above bars indicate significant differences across conditions (one-sample two-sided bootstrap test, **: $p < 0.01$; *: $p < 0.05$; FDR-corrected). Source data are provided as a Source Data file.

**Supplementary Note 2: Influence of low-pass temporal filtering on discrimination onsets**

To test whether our choice of low-pass filtering (30 Hz) affected the onset latencies by potentially shifting or blurring information, we ran the same image decoding and RSA analyses (Fig. 2a and 2b) as described in the main text but on unfiltered data. The analyses of unfiltered data resulted in highly similar onset latencies as in the case of filtered data. Individual images could be discriminated by visual representations from 53 ms onwards (49 ms for filtered data; Supplementary Fig. 2a; all cluster-corrected sign permutation tests; cluster-defining threshold $p < 0.05$; corrected significant level $p < 0.05$). For face dimensions (Supplementary Fig. 2b), visual MEG representations were able to discriminate faces by age starting at 68 ms (61 ms for filtered data), gender at 81 ms (72 ms for filtered data) and specific identity at 96 ms (91 ms for filtered data). While the latencies for gender, age and identity show a trend towards later onsets for unfiltered data, this difference was minimal (around 5 – 9 ms). However, we found a large shift in onset latency for the discrimination of familiarity (583 ms versus 403 ms for filtered data). This effect can be explained by the fact that unfiltered data are noisier which affects the power to detect small effects, such as the familiarity effect found here. Overall, we conclude that the early onset latencies for gender, age and identity reported in our main analysis are only minimally affected by the use of a low-pass filter.

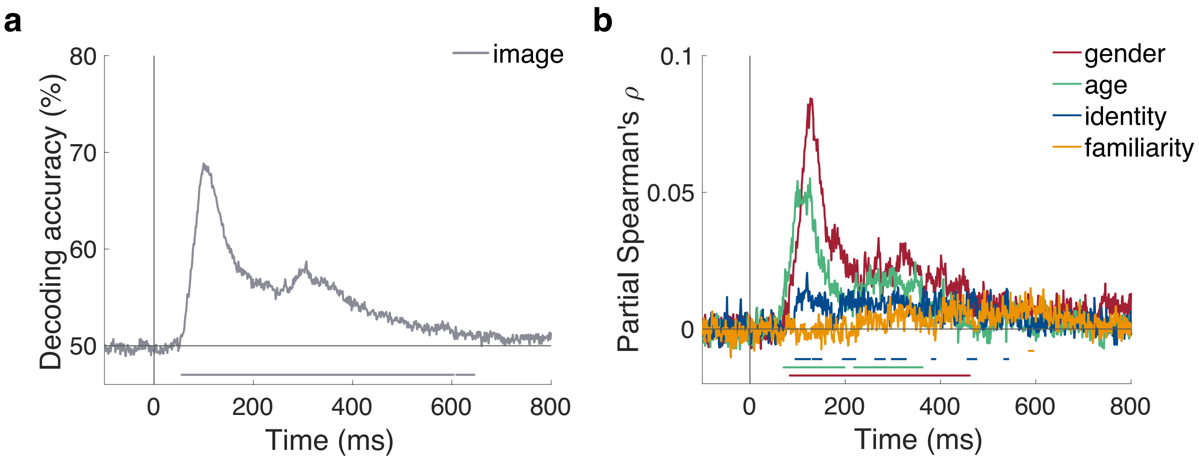


**Supplementary Figure 2 | Decoding of face dimensions from unfiltered MEG signals.** (**a**) Time course of image decoding where 0 indicates image onset (n = 16). **(b)** Time course of partial Spearman correlations between MEG RDMs and model RDMs (see Fig. 1c) for gender (red), age (green), identity (blue) and familiarity (orange), partialling out all other models and low-level features (see Methods). Lines below plots indicate significant times using cluster-based sign permutation test (cluster-defining threshold $p < 0.05$, and corrected significance level $p < 0.05$). Source data are provided as a Source Data file.

**Supplementary Note 3: Cross-decoding of face dimensions from MEG signals**

**Methods.** We used RSA as our main analysis (see Methods in the main manuscript) to analyze the representation of face dimensions in the MEG data. An alternative approach to measure categorical information while controlling for irrelevant dimensions is to use “cross-decoding” by training a classifier on a face dimension (e.g., gender) using one set of stimuli (e.g., a subset of identities), while testing on the left-out set of stimuli (e.g., the left-out identities). The decoding accuracy then gives an estimate of the amount of category information invariant to the cross-validated condition. To get an estimate of the decoding accuracies for the different face dimensions and to test whether the time course of cross-decoding is similar to the time course of RDM-based model correlations, we additionally performed a cross-validated classifier analysis. This classification analysis was performed separately for each subject in a time-resolved manner. As for the model-based approach, a pattern in the analysis consisted of the principal component scores for one trial and one condition at a given time point, and we used linear support vector machines (SVM; libsvm [2]) as classifiers. For gender, age and familiarity, we trained the classifier in a leave-one-identity out scheme. In addition, we controlled for the other irrelevant dimensions by balancing these dimensions across training and testing. For example, when training a classifier on gender, we selected one female and one male identity for testing that were both either old or young and either familiar or unfamiliar identities. This was necessary to avoid any bias in the classifier towards an irrelevant dimension (e.g., in case the test set would consist of a young female and an old male identity, female identities in the training would more likely be older than male identities and thus bias the classifier towards classifying the young female identity as male during testing). For gender, age and familiarity, this resulted in 32 combinations to select two of the 16 identities for testing. For each of these combinations, we trained the classifier on groups of trials by averaging all trials of the training data for each condition into seven folds. We then tested the classifier on the average of all trials of the testing data for each condition (i.e., corresponding to an eight-fold cross-validation). This classification procedure was repeated 100 times. The average across the 32 identity combinations and the 100 repetitions per combination served as decoding accuracy for this dimension at a specific time point. For identity classification, we used a different decoding scheme. To obtain classification accuracy for identity, we trained a classifier on each of the eight possible identity pairs of the same gender, age and familiarity and each of the 25 combinations to choose two images for testing. For each of these combinations (8 x 25 in total), we trained a classifier on groups of trials by averaging all trials of the training images into four folds, and tested on the average of the testing images (i.e., 5-fold cross-validation). This

procedure was repeated 100 times. For each time point, we computed the decoding accuracy for identity by averaging across the 200 identity and image combinations and the 100 repetitions. This procedure resulted in four decoding accuracy time courses for each subject which we then tested for statistical significance using the same permutation-based cluster-size inference as for the RSA correlation time courses in the main manuscript.

**Results.** Our cross-decoding analysis of the time course of information about each of the face dimensions produced results highly similar to our RSA analysis. In particular, we were able to decode all face dimensions above chance (Supplementary Fig. 3). Similar to the RSA analysis, the highest decoding accuracy was found for gender (peak decoding accuracy 77% at 136 ms after stimulus onset), followed by age (73% at 106 ms), and then identity (57% at 115 ms). In line with our RSA analysis, age (57 – 419 ms; all cluster-corrected sign permutation tests; cluster-defining threshold $p < 0.05$; corrected significant level $p < 0.05$) and gender (68 – 613 ms; 718 – 779 ms) were decodable first from MEG representations, followed by identity (80 – 245 ms; 293 – 342 ms; 358 – 408 ms). We further found a late signature of familiarity (peak decoding accuracy 59% at 490 ms; significant time points: 480 – 530 ms). Note that there is a later peak for familiarity at 606 ms that did not reach significance. Overall, we conclude that the time course of cross-decoding is similar to the results obtained with the RSA analysis.

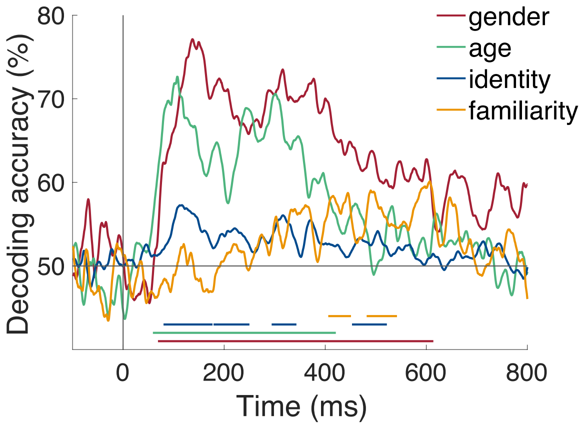


**Supplementary Figure 3 | Cross-decoding of face dimensions from MEG signals.** Time course of cross-decoding accuracy for gender (red), age (green), identity (blue) and familiarity (orange) (n = 16). Gender, age and familiarity classifier training and testing was performed in a leave-one-identity out decoding scheme, whereas identity training and testing was performed in a leave-one-image out decoding scheme. Source data are provided as a Source Data file.

**Supplementary Note 4: Temporal generalization of MEG multivariate pattern analysis**

**Methods.** To get a measure of how well neural representations generalized across time, we extended the multivariate pattern analysis by a temporal generalization approach [3-5]. Based on this approach, a classifier is trained on the data of a specific time point *t* (training time) and tested on all other time points *t'* (testing time). Intuitively, if representations are stable over time, a classifier should be able to discriminate two conditions not only at the trained time, but also at later time points. Note however, that a lack of stability could theoretically also be due to instability of noisy or irrelevant signals. Given how established this approach is, and considering the range of different patterns of generalization which have been found across different tasks (for a review see [3]), this is unlikely. To test generalization of neural representation across time for our face stimuli, we used the same classification scheme as used for classification on identical time points. However, to save computation time we used a cross-validated pairwise correlation classifier instead of a linear support vector machine (SVM). This classifier reached slightly lower decoding accuracy values when trained on identical time points (i.e., the diagonal values in Supplementary Fig. 4) than the SVM (c.f., Fig 2a) but a highly similar pattern over time. Similar to the classification analysis at identical time points, we performed temporal generalization analysis separately for each subject and averaged the decoding accuracies across stimulus pairs. This yielded a 901 x 901 matrix (-100 to 800 ms with respect to stimulus onset) for each subject.

**Results.** We found that neural representations were sustained for a short amount of time (up to around 200 ms), and quickly decreased below chance for larger time spans (Supplementary Fig. 4). Surprisingly, we found a lack of generalization very early between around 100 and 200 ms (shown as non-significant off-diagonal areas between 100 and 200 ms), with a reactivation of the neural representations at later stages. This could reflect some early feedback or recurrent processing. Overall, we found that neural representations are mainly transient and do not persist over time, suggesting continuous transformations of stimulus information.

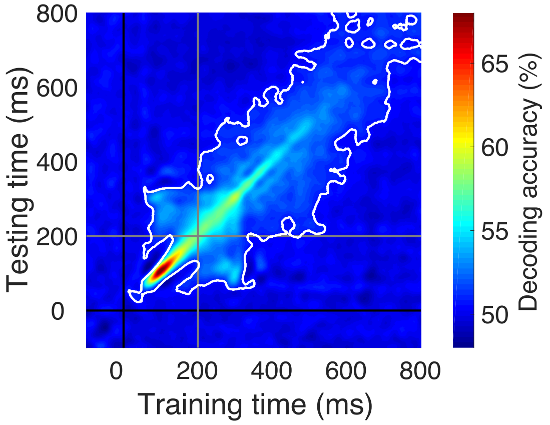


**Supplementary Figure 4 | Temporal generalization of face image decoding.** The correlation classifier trained with principal components extracted from all MEG sensors at time point *t* (training time) and tested on all other time points (testing time). The temporal generalization decoding matrix was averaged over all image pairs and all subjects (n = 16), thus corresponding to the temporal generalization of the image decoding time course shown in Fig. 2a. Black line marks image onset, and the gray line marks the image offset. The white contour indicates significant decoding values using one-sided cluster-based sign permutation test (cluster-defining threshold $p < 0.05$, and corrected significance level $p < 0.05$). Source data are provided as a Source Data file.

## Supplementary References

1. O'Toole, A. J., Castillo, C. D., Parde, C. J., Hill, M. Q. & Chellappa, R. Face Space Representations in Deep Convolutional Neural Networks. *Trends Cogn. Sci.* **22,** 794–809 (2018).
2. Chang, C.-C. & Lin, C.-J. LIBSVM: A library for support vector machines. *ACM Trans. Intell. Syst. Technol.* **2,** 27 (2011).
3. King, J.-R. & Dehaene, S. Characterizing the dynamics of mental representations: the temporal generalization method. *Trends Cogn. Sci.* **18,** 203–210 (2014).
4. Isik, L., Meyers, E. M., Leibo, J. Z. & Poggio, T. The dynamics of invariant object recognition in the human visual system. *J. Neurophysiol.* **111,** 91–102 (2014).
5. Cichy, R. M., Pantazis, D. & Oliva, A. Resolving human object recognition in space and time. *Nat. Neurosci.* **17,** 1–10 (2014).
